# Holistic Approach to Tuberculosis Detection, Treatment and Prevention: Emerging Evidence and Strategies from the Field

**DOI:** 10.3390/tropicalmed7030036

**Published:** 2022-02-25

**Authors:** Abu Naser Zafar Ullah, Sourya Shrestha, Amyn A. Malik, Tapash Roy

**Affiliations:** 1 Department of Public Health, Daffodil International University, Daffodil Smart City, Ashulia, Dhaka 1341, Bangladesh; 2 KIT Royal Tropical Institute, 64 Mauritskade, 1092 AD Amsterdam, The Netherlands; 3 Department of Epidemiology, Bloomberg School of Public Health, Johns Hopkins University, 615 N. Wolfe Street, Baltimore, MD 21205, USA; 4 Yale Institute for Global Health, Yale University, New Haven, CT 06510, USA; 5 IRD Global, Singapore 238884, Singapore; 6 Interactive Research and Development, Bangladesh (IRD Bangladesh), Gulshan 1, Dhaka 1212, Bangladesh

The global fight against tuberculosis (TB) has gained momentum since the adoption of the ‘End TB Strategy’ in 2014 [1], and the inclusion of ‘ending the TB epidemic’ as a target within the Sustainable Development Goals (SDGs) 3 in 2015 [1,2]. During the past two decades, improvements in quality-assured diagnosis and treatment of TB has contributed to saving more than 40 million lives and reducing TB mortality rates by more than 40% globally [3]. However, we have not been as successful in reducing TB incidence rates: TB incidence is declining by 2% per year globally, far behind the pace necessary to meet the End TB targets [1,3]. Therefore, adopting a holistic approach that combines interventions that are biomedically oriented or focused on social protection, and that are tailored to regional, national and local contexts, is required. As such, countries will need to strengthen their health and social sectors (e.g., by making progress towards achieving universal health coverage, or increasing social protection), as emphasized within the framework of the new SDG agenda [2]. Yet, all this may still not be enough to end the TB epidemic, and will further require not just innovation of novel tools, but also innovative ways of using existing tools, that is shaped by emerging evidence from the field. This Special Issue provides an opportunity to publish the compelling evidence right from the field level in regard to the essentials of operationalizing the principles, pillars and components of the End TB Strategy and the United Nations High-Level Meeting (UNHLM) on TB.

The progress toward reducing TB burden, which was already lagging behind the SDG targets, was further disrupted by the coronavirus disease 2019 (COVID-19) pandemic. The pandemic has resulted in sharp reductions in TB notification rates globally, which is projected to result in 6.3 million additional TB patients and 1.4 million TB deaths by 2025 [4]. However, a study on this issue by Malik and colleagues [5] shows that such disruptive challenges can be met head-on, by taking an adaptive and collaborative approach. The authors demonstrate that a collaborative effort by the provincial TB program and private sector partners, and integration of the key activities associated with diagnosis and linkage to care of TB and COVID-19 have effectively managed to bring TB notification rates up to 90% of expected levels in Karachi, Pakistan.

Treatment success rates for drug-resistant TB, and specifically Rifampicin-resistant TB (RR-TB), remains low: not only do RR-TB patients have high morbidity, but such poor treatment outcomes also increase the risk of further transmission. Reuter et al. [6] reported the outcomes of implementing a substance-use screening program for people living with RR-TB in South Africa. In this retrospective study, the authors show that substance use is common among persons with RR-TB, a finding that is largely consistent with previous studies. The authors argue that there is a need for interventions to address this co-morbidity as part of “person-centered care”. Integrated, holistic care is also needed at the community level to address unique challenges of persons with RR-TB and Substance use.

Reactivation of latent tuberculosis infection (LTBI) or latent parasitic infection (LPI) during drug-induced immunosuppression can have serious consequences. Carnino et al. [7] sought to determine the seroprevalence of LTBI and LPI in such patients at the Geneva University Hospitals in Switzerland and explore its relationship with country of origin or previous travel. They argue that screening before immunosuppressive therapy needs to be individualized, and LTBI and LPI need to be ruled out in patients who originate from or have travelled to high-prevalence countries. The sensitivity of strongyloidiasis serology is reduced following immunosuppression, so an algorithm combining different tests or presumptive treatment should therefore be considered for further implementation.

Globally, a substantial proportion of people who complete anti-tuberculosis treatment experience significant morbidity and mortality, which can negatively affect their quality of life. It is therefore crucial for National TB Programs to conduct end-of-treatment assessments. This study conducted across four provinces in China by Lin and colleagues [8] shows that it is feasible to conduct post-TB assessments under routine programmatic conditions (requiring only 20 min of TB program staff’s time on average), and may significantly reduce the morbidity among people who had completed anti-TB treatment (since about half of the individuals screened had ongoing symptoms).

Successful implementation of child contact screening and management has proven to be difficult, reflected in the fact that there are few field examples from TB-endemic countries. In India, the challenges in pediatric TB contact screening and chemoprophylaxis initiation are also underexplored. A study from India (Chawla et al. [9]) explored a comprehensive approach to understand the challenges in contact screening and chemoprophylaxis initiation and implementation of the pediatric household contacts as perceived by health care providers. Key challenges identified included stigma, difficulty reaching patients and collecting samples, and difficulty ensuring acceptance and adherence of TB preventive treatment (TPT). These findings provide the National TB Program with specific gaps to address. There is a growing evidence that the childhood TB is consistently under-detected and TPT for high-risk child contacts is poorly implemented in most high-burden countries. An integrated Child TB Project implemented in Uganda (Dongo et al. [10]) resulted in improved case finding of child and adult TB cases, improved treatment outcomes for child TB and high uptake and completion of TPT for eligible child contacts. 

Moreover, Zhao et al. [11] demonstrated that telehealth can offer an alternative option for follow up visits and thereby improve treatment adherence among children with TB infection.

Nagaraja et al. [12] analyzed the National Tuberculosis Elimination Program data of Active Case Finding (ACF) intervention implemented in India and revealed that the Indian states that tested a greater proportion of those were screened during ACF and used chest X-rays or Cartridge Based Nucleic Acid Amplification Test (or both) to diagnose TB had a higher diagnostic yield with a lower number needed to screen to diagnose one person with TB. High proportion of TB-affected households experience catastrophic costs, and there is arguably a need for TB-specific social protection programs in patient-centered healthcare. In this context, Aung et al. [13] conducted a survey using the WHO methodology reported that 60% of TB-affected households face catastrophic costs (where the costs are >20% of the annual household income) in Myanmar; and these findings have led the government and donors to increase support for multidrug-resistant tuberculosis (MDR TB) patients. The significant proportion of total spending attributable to lost income and food or nutritional supplements suggests that income replacement programs and/or food packages may ameliorate the burdensome costs.

It is increasingly clear that there is no silver bullet to end the TB epidemic. This is partly because the challenges associated with TB epidemic are multi-faceted, deep rooted, but also changing, as highlighted in this issue ranging from dealing with new disruptive forces such as the pandemic to old ones such as stigma and barriers to access; and from addressing TB in pediatric and drug resistant forms to dealing with morbidity in post-TB life. While ensuring that research and innovation are thriving, it is also equally important to focus on implementation of a mix of biomedical, public health and socioeconomic interventions. These studies also highlight that collaboration and communication between the private sector, health services and national tuberculosis control programs must be strengthened. Integration with the wider health sector is critical, as it has been recognized as an important part of the global tuberculosis control strategy beyond 2015. We have many of the tools we need to implement the necessary TB programs, and we have made much progress gathering data and evidence to identify policies that are likely to be impactful; perhaps even the political will is also beginning to emerge.

## Data Availability

Not applicable.
